# The effects of selective serotonin reuptake inhibitors on memory functioning in older adults: A systematic literature review

**DOI:** 10.1177/02698811221080462

**Published:** 2022-04-29

**Authors:** Julie EM Schulkens, Kay Deckers, Maud Jenniskens, Arjan Blokland, Frans RJ Verhey, Sjacko Sobczak

**Affiliations:** 1 Department of Psychiatry and Neuropsychology, School for Mental Health and Neuroscience (MHeNs), Alzheimer Centre Limburg (ACL), Maastricht University, Maastricht, The Netherlands; 2 Department of Old Age Psychiatry, Mondriaan Hospital, Heerlen, The Netherlands; 3 Department of Neuropsychology and Psychopharmacology, Faculty of Psychology and Neuroscience, Maastricht University, Maastricht, The Netherlands

**Keywords:** Selective serotonin reuptake inhibitor, antidepressants, memory, cognition, older adults, aging, pharmacotherapy

## Abstract

**Introduction:**

Selective serotonin reuptake inhibitors (SSRIs) are commonly prescribed to older adults. In contrast to young subjects, it is unclear whether older adults may be vulnerable to cognitive side effects. Serotonin is involved in cognitive functions (e.g. memory). It is of great importance to examine the effects of SSRIs on memory functioning in older adults.

**Objectives:**

The objective of this systematic literature review is to summarize studies in which the effects of SSRI treatment on all aspects of memory functioning in older adults are investigated.

**Methods:**

PubMed, PsycINFO, CINAHL, and Embase were searched for all studies published until 18th of October 2021. Articles were included if they fulfilled the inclusion criteria as follows: (1) study design is (randomized) controlled trial, cross-sectional, or prospective cohort study; (2) study population consists of older adults (mean age ⩾65 years), or results for this age-group are reported separately; (3) intervention is use of an SSRI; and (4) effects on performance of any memory domain are measured and clearly described.

**Results:**

The search yielded 1888 articles, of which 136 were included for the full-text review. Eventually, 40 articles were included. Most studies reported no association between SSRI use and memory functioning. The studies that found a positive association mainly investigated older adults with mental or neurological disorders (e.g. depression or stroke). A few studies found a negative association in the following subgroups: non-responders (depression), patients with frontal brain disease, and women.

**Conclusion:**

Overall, no consistent negative effects of SSRIs on memory functioning in older adults were found after SSRI treatment. Most studies reported no change in memory functioning after SSRI use. Some studies even showed an improvement in memory performance. Positive effects of SSRIs on memory functioning were especially found in older adults with mental or neurological disorders, such as subjects with depression or stroke.

## Introduction

Over the last decade, the use of antidepressants seems to be rising (Brett et al., 2017). A longitudinal study observed that 6.6% of community-dwelling older adults used a selective serotonin reuptake inhibitor (SSRI) at 6 years follow-up (Marcum et al., 2016). In older adults, indications for antidepressant treatment are the same as they are for younger adults, for example, depression and anxiety disorders (Kok, 2013). It is well known that depression is often associated with memory impairment, both in adults and older adults (Marazziti et al., 2010). Remission of depression can lead to improvement of cognitive functioning (Story et al., 2008), suggesting a relation between depressive symptoms and cognitive functions. However, it has also been described that cognitive recovery can occur independent of depression treatment response (Jorge et al., 2010). This finding indicates that antidepressant treatment may have a dissociable effect on depressive symptoms and cognition.

Prevalence of cognitive impairment increases with age and is associated with lower quality of life (Bárrios et al., 2013). Therefore, it is relevant to investigate the effect of commonly prescribed medication, such as SSRIs, on cognitive functioning in older adults. It is plausible to assume that SSRIs affect cognitive functions, since serotonin (5-HT) is involved in attention and (working)memory (Štrac et al., 2016). Studies in older adults are lacking, but previous serotonergic challenge studies have suggested that SSRI administration is associated with acute improvement of long-term memory (Harmer et al., 2002) and positive information processing (Harmer et al., 2004; Norbury et al., 2009) in healthy adult volunteers. However, other studies have observed no acute effect on cognitive functioning (Rose et al., 2006; Siepmann et al., 2003) or even suggested impairment of vigilance after sub-chronic treatment (Riedel et al., 2005).

With aging, changes in 5-HT functioning occur; receptor expression and binding affinity of multiple 5-HT receptors have been shown to decrease in older adults (Fidalgo et al., 2013; Lu et al., 2004). Impaired 5-HT neurotransmission in aging has been associated with impaired cognitive processes (Rodriguez et al., 2012). Based on these changes, it could be hypothesized that SSRI treatment in older adults may have a different effect than in younger adults.

In conclusion, SSRIs are commonly prescribed to older adults, who already seem vulnerable to memory impairment due to age-related serotonergic changes and neurodegeneration. Therefore, it is important to know the possible effects of SSRIs on memory functioning in older adults. Given the limited amount of studies conducted in older adults, there is a need for a broad and general synthesis of the evidence. The aim of this systematic literature review is therefore to summarize all relevant findings on the effects of SSRIs on all aspects of memory function in older adults.

## Methods

### Protocol and registration

This systematic review was registered in the PROSPERO database under the ID CRD42021283326.

### Data sources and search

The literature search was conducted in databases PubMed, PsycINFO, CINAHL, and Embase. The search consisted of predictor-related terms (e.g. SSRI), outcome-related terms (e.g. short-term memory and cognition), and specific limitations (e.g. humans and language restrictions). The complete search strategy is provided in Supplemental Appendix 1.

### Study selection

All publications until the 18th of October 2021 were included if they fulfilled the following eligibility criteria: (1) study design is (randomized) controlled trial, cross-sectional, or prospective cohort study; (2) study population consists of older adults (mean age ⩾65 years), or results for this age-group are reported separately; (3) intervention is use of an SSRI; and (4) effects on performance of any memory domain, such as global memory (assessed by the Wechsler Memory Scale (WMS), mini-mental state examination (MMSE), etc.), semantic memory, visual/spatial memory, working memory, episodic memory, long-term memory, and/or short-term memory are measured and clearly described. Reference lists of publications and secondary literature (review articles, editorials, book chapters, etc.) were hand-searched for possible missing articles.

### Data extraction and quality assessment

The selection process was in accordance with the Preferred Reporting Items for Systematic Review and Meta-Analyses (PRISMA) guidelines (Moher et al., 2009). Two authors (J.E.M.S. and S.S.) independently screened titles and abstracts for potential eligibility based on the abovementioned eligibility criteria. Next, full-text articles of potentially relevant citations were examined by these two authors (J.E.M.S. and S.S.). Disagreements were resolved by discussion until consensus was reached.

A standardized data collection form was used to extract information. Information was extracted from each included publication on: (1) study design; (2) characteristics of participants, including age, disease and use of co-medication; (3) type of intervention, including type of SSRI, dosage and duration of the intervention, and, if applicable, intervention for the control group; (4) tests used to assess memory performance; (5) observed effect of treatment on memory. One author (M.J.) extracted the data from included full-text articles and one author (J.E.M.S.) checked the extracted data. If full-text articles were not available, corresponding authors and/or affiliated institutes were contacted by e-mail.

The Cochrane Collaboration Tool for Risk of Bias (Higgins et al., 2011) was used for the quality assessment of randomized controlled trials. The adapted version for cross-over trials (Sterne et al., 2019) was used for randomized cross-over trials. Non-randomized clinical trials were assessed using the Risk of Bias In Non-randomized Studies of Interventions (ROBINS-I) (Sterne et al., 2016). Assessment of risks of bias and quality was done by author J.S.

### Synthesis of result

Data will be presented in a narrative manner, because heterogeneous results inherent to the broad research question will make a meta-analytic analysis less appropriate. First, we will present the general results of SSRI treatment on memory functioning per memory domain. Second, we will present results for various populations of older adults (e.g. with depression and stroke).

## Results

**Figure 1.**
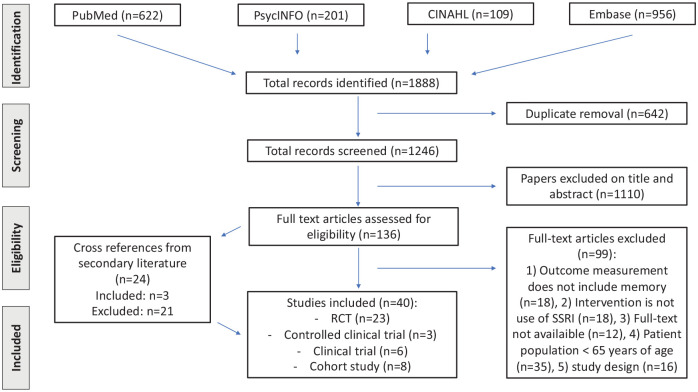
PRISMA flowchart search and screening process.

The search yielded 1888 articles, of which 136 were included for full-text review. Of these, 99 were excluded due to different reasons based on the exclusion criteria (Figure 1). Twenty-four potentially relevant articles were found from cross-references, of which three were included. We contacted 15 authors to obtain full-text articles that were not available to us. Of these, three authors responded. Eventually, a total of 40 articles were included, of which 23 randomized controlled trials, 3 controlled clinical trials, 6 non-controlled clinical trials, and 8 prospective cohort study. All 40 studies and their details and results are summarized in Table 1.

**Table 1.** Characteristics of included studies.

Author (year)	Subjects	Age in years, mean (SD), range	Intervention (*n*)	Duration of treatment	Memory measure	Main objectives and findings
		Randomized controlled trial*Depression*				
Boggio et al. (2005)	Patients with Parkinson’s disease and minor or major depression (mean HAM-D 24.5)	65.2 (1.65)	Fluoxetine 20 mg + sham rTMS (*n* = 12) rTMS + placebo (*n* = 13)	8 weeks	Digit span forward (DS-f) and backward (DS-b)	Objective: to compare the cognitive effects of fluoxetine and rTMS in PD patients. Finding: executive cognitive function improved after fluoxetine and rTMS.
Bondareff et al. (2000)	Single episode or recurrent MDD (DSM-III-R) (mean HAM-D 24.7)	67.8 (6.0) 67.9 (6.6)	Sertraline 50–150 mg (*n* = 105) Nortriptyline 25–100 mg (*n* = 105)	12 weeks	MMSE, DSST, SLT	Objective: to evaluate the comparative efficacy and safety of sertraline and nortriptyline for the treatment of MDD in older adults. Finding: advantage for sertraline in cognitive function.
Cassano et al. (2002)	MDD (ICD-10) (mean HAM-D 23.5)	75.4 (6.6)	Paroxetine 20–40 mg (*n* = 123) Fluoxetine 20–60 mg (*n* = 119)	1 year	MSSE, WRT, Blessed Information and Memory Test, the Clifton Assessment Schedule	Objective: to evaluate the effect of paroxetine and fluoxetine on cognitive functions and depressive symptoms in non-demented depressed elderly in the long-term. Finding: both were effective, safe, and well tolerated and some improvement in the majority of cognitive test was observed.
Culang et al. (2009)	Single or recurrent non-bipolar, non-psychotic MDD (DSM-IV) (mean HAM-D 24.3)	79.6 (4.4)	Citalopram 20 mg (*n* = 84) Placebo (*n* = 90)	8 weeks	WRT	Objective: to examine the impact of antidepressant treatment on change in cognitive functioning. Finding: the citalopram group improved on some tests but declined on others, the pattern of change depended on responder status.
Geretsegger et al. (1994)	In- and out-patients with MDD (DSM-III) (mean HAM-D 28.5)	74.3, 61–85 73.7, 65–85	Paroxetine 20–40 mg (*n* = 54) Fluoxetine 20–60 mg (*n* = 52)	6 weeks	MMSE	Objective: to compare the antidepressant efficacy of paroxetine in the treatment of elderly depressed patients with that of fluoxetine and their effects on cognitive and behavioral function. Finding: both SSRIs improved symptoms of depression and cognitive function, but paroxetine showed superiority over fluoxetine
Newhouse et al. (2000)	Outpatients with MDD (DSM-III) (mean HAM-D 25.1)	68 (5.3) 67 (5.9)	Sertraline 50–100 mg (*n* = 117) Fluoxetine 20–40 mg (*n* = 119)	12 weeks	SLT, DSST	Objective: to compare efficacy and safety of sertraline and fluoxetine in elderly depressed patients and to compare cognitive functioning. Finding: sertraline and fluoxetine had comparable efficacy, but sertraline produced a greater improvement in cognitive performance
Raskin et al. (2007)	Recurrent MDD (DSM-IV) (mean HAM-D 22.4)	72.6 (5.7) 73.3 (5.7), 65–90	Duloxetine 60 mg (*n* = 207) Placebo (*n* = 104)	8 weeks	WRT, DSST, letter–number sequencies test (WAIS), MMSE	Objective: to compare the effects of duloxetine versus placebo on cognition, depression, and pain in elderly patients with MDD. Finding: duloxetine treatment resulted in improvement in cognitive functions, mainly verbal learning and memory.
Taragano et al. (1997)	Outpatients with MDD (DSM-III) and probable AD (NINCDS/ADRDA criteria) (mean HAM-D 25.8, mean MMSE 19.4)	71.7 (5.0) 72.4 (4.9)	Fluoxetine 10 mg (*n* = 18) Amitriptyline 25 mg (*n* = 19)	6 weeks	MMSE	Objective: to compare the efficacy of amitriptyline and fluoxetine on depression outcome, cognitive performance, and safety in elderly patients with MDD and probable AD. Finding: changes in MMSE score were not substantially different between baseline and follow-up in both fluoxetine and amitriptyline-treated groups.
		*Dementia*				
Choe et al. (2016)	Patients with probable AD (mean MMSE 16.9)	74.33 (7.12) 74.82 (9.19)	Escitalopram 20 mg (*n* = 28) Placebo (*n* = 29)	1 year	ADAS, MMSE	Objective: to investigate whether escitalopram would decelerate the brain atrophy of patients with mild-to-moderate AD. Finding: intervention with escitalopram did not slow the progression of hippocampal or whole brain atrophy compared with placebo.
Deakin et al. (2004)	FTD (clinical diagnosis) (mean MMSE 23.2)	66.3 (6.9), 54–75	Paroxetine 40 mg Placebo (cross-over, *n* = 10)	4 weeks	DS-f, DS-b, CANTAB	Objective: to assess the effect of paroxetine on performance on tests of ventral lobe function. Finding: paroxetine did not improve scores on any measure.
Mokhber et al. (2014)	Patients diagnosed with mild to moderate AD (mean MMSE 15)	67.3 (3.0) 67.9 (2.8) 67.6 (5.3)	Sertraline 150 mg (*n* = 20) Venlafaxine 150 mg (*n* = 20) Desipramine 150 mg (*n* = 19)	12 weeks	MMSE	Objective: to compare the efficacy of sertraline, venlafaxine, and desipramine on depression, cognition, and the activities of daily living in Alzheimer’s patients. Finding: sertraline improved depression, cognition, and daily functioning significantly; venlafaxine improved cognition and daily functioning; desipramine only improved daily functioning.
Munro et al. (2004)	Outpatients with AD (NINCDS/ADRDA criteria) and MDD (DSM-IV) (median MMSE 21 mean depression severity N/A)	75.5 (9.7) 79.6 (5.2)	Sertraline 50–150 mg (*n* = 24) Placebo (*n* = 20)	12 weeks	MMSE, WRT, RBMT	Objective: to determine the cognitive effects of depression reduction in AD patients and to investigate the cognitive effects of sertraline in these patients. Finding: there was no evidence that treatment with sertraline was associated with improved cognitive functioning.
Munro et al. (2012)	AD with clinically significant depressive symptoms (DSM-IV) (mean MMSE 17.7, mean HAM-D 22.7)	76.5 (8.0) 78.2 (8.0),	Sertraline 100 mg (*n* = 67) Placebo (*n* = 64)	24 weeks	MMSE, DS-b, ADAS	Objective: to study cognitive outcomes in patients with AD and depressive symptoms. Finding: there was no effect of treatment or depression remission on cognitive test performance.
Porsteinsson et al. (2014)	Probable AD (mean MMSE 15.7) and clinically significant agitation	78 (9) 79 (8)	Citalopram 30 mg (*n* = 94) Placebo (*n* = 92)	9 weeks	MMSE	Objective: to evaluate the efficacy of citalopram for agitation in patients with Alzheimer’s disease and without major depression. Finding: citalopram treatment led to a reduction in agitation in patients with Alzheimer’s disease.
Weintraub et al. (2010)	Outpatients with AD (DSM-IV) and depression of AD (mean MMSE 20.3, mean depression severity N/A)	Median age = 79 years	Sertraline 50–100 mg (*n* = 67) Placebo (*n* = 64)	24 weeks	MMSE	Objective: to investigate any delayed benefits of sertraline that are the result of sustained depression reduction. Finding: sertraline treatment was not associated with long-term benefits in mood, neuropsychiatric symptoms, function, quality of life, or global cognition in patients with AD and depression of AD.
		*Alcohol-related disorders*				
Martin et al. (1989)	Alcohol amnestic disorder (mean WMS score 89.5)	66 (2)	Fluvoxamine 100–200 mg (*n* = 6)	4 weeks	WMS, WRT	Objective: to study neurochemical and cognitive effects of fluvoxamine in patients with chronic organic mental disorders associated with alcoholism. Finding: treatment with fluvoxamine improved episodic memory for patients with alcohol amnestic disorder.
O’Carroll et al. (1994)	Korsakoff’s syndrome (mean CAMCOG 69.6)	69.3 (4.1)	Fluvoxamine 200 mg Placebo (cross-over, *n* = 11)	4 weeks	WMS, consonant trigrams, DS-f, DS-b, RBMT	Objective: to study whether cognitive functions are affected by fluvoxamine in patients with Korsakoff’s syndrome. Finding: no cognitive enhancing effect was found, but verbal fluency worsened after treatment.
		*Other medical conditions*				
Butters et al. (2011)	Outpatients with late-life generalized anxiety disorder (mean HAM-A 22.8) who scored below the total group median RBANS score of 94	71.6 (7.7)	Escitalopram Placebo Total *n* = 66	12 weeks	RBANS DS, immediate memory, delayed memory, and letter–number sequencing	Objective: to characterize neuropsychological function among older adults with GAD and to identify any changes related to treatment of anxiety. Finding: older participants with GAD performed worse than the comparison group on information processing working memory, inhibition and problem-solving, as well as immediate and delayed memory. Those who received citalopram had greater improvements on problem-solving, concept formation, and mental flexibility.
Jorge et al. (2010)	Three months post-stroke	64.2 (13.9) 60.8 (14.4) 68.9 (11.7), 50–90	Escitalopram 5–10 mg (*n* = 43) Placebo (*n* = 45) PST (*n* = 41)	12 months	RBANS	Objective: to examine the effect of escitalopram on cognitive outcome in stroke patients. Finding: compared to patients receiving placebo or PST, patients receiving escitalopram showed better global functioning and specifically verbal and visual memory.
Robinson et al. (2000)	Acute stroke, both non-depressed and depressed patients (HAM-D ⩾12)	65 (14) 73 (8)	Fluoxetine 40 mg (*n* = 40) Nortriptyline 100 mg (*n* = 31) Placebo (*n* = 33)	12 weeks	MMSE	Objective: to examine the effects of antidepressants on recovery of post-stroke patients and whether an effect is independent of depression recovery. Finding: there was no effect of medication on recovery in activities of daily living, cognitive function, or social functioning.
		*General population*				
Furlan et al. (2001)	Healthy older adults	70.7 (3.5) 75.1 (6.4) 70.3 (4.9)	Sertraline 150 mg (*n* = 12) Paroxetine 40 mg (*n* = 18) Placebo (*n* = 19)	3 weeks	WRT, DSST, paired-associate learning task	Objective: to study whether paroxetine demonstrates impairment on cognitive tasks compared to sertraline and placebo. Finding: no significant differences in effects on performance between groups.
Kerr et al. (1992)	Healthy older adults	N/A, 60–85	Paroxetine 20 mg Lorazepam 1 mg Placebo (cross-over, *n* = 15)	2 weeks	Sternberg Scanning	Objective: to examine the effects of paroxetine, alone and in combination with alcohol, on psychomotor function and cognitive function in healthy older volunteers. Finding: paroxetine had little or no effect on most of the test variables, but improved information processing ability.
Lenze et al. (2020)	Community-dwelling older adults with subjective cognitive decline, scoring within one SD of age-matched mean score of cognition battery	71.68 (4.77) 71.88 (5.30)	Vortioxetine 10 mg + cognitive training (*n* = 51) Placebo + cognitive training (*n* = 49)	26 weeks	NIH Toolbox Cognition Battery (i.e. List Sorting Memory Test, Picture Sequence Memory Test)	Objective: to test the efficacy of vortioxetine added to a cognitive training program to remediate age-related cognitive decline. Finding: the combination of vortioxetine with computerized cognitive training for showed greater improvement in global cognitive performance compared with cognitive training with placebo.
		Controlled clinical trial				
		*Depression*				
Alves et al. (2007)	Heart failure patients without or with MDD (*DSM*-IV, mean HAM-D 27.4)	74.6 (5.6)	Citalopram 20 mg (*n* = 13) Sertraline 50 mg (*n* = 5) None (*n* = 41)	8 weeks	CAMCOG	Objective: to assess the impact of depressive symptoms on patterns of cognitive deficits associated with heart failure and the impact of antidepressant treatment. Finding: improved total CAMCOG and remote memory scores, but no effect on recent memory scores in heart failure patients with MDD after SSRI treatment.
Beheydt et al. (2015)	In- and out-patients with unipolar single episode or recurrent MDD (DSM-IV-TR, mean GDS 17.6) and healthy controls	74.7 (7.6)	Escitalopram 5–20 mg (*n* = 28) None (*n* = 20)	12 weeks	WRT, DSST	Objective: to investigate the differential effects of escitalopram on cognitive and psychomotor measures in elderly patients and compare them to mood effects. Finding: treatment improved mood to a moderate level, but cognitive and motor functioning changed much less.
Savaskan et al. (2008)	Inpatients with major depression, first episode (ICD-10, mean GDS 9.4) compared to healthy age-matched controls	76.2 (1.8)	Escitalopram 5–20 mg (*n* = 18) None (*n* = 22)	4 weeks	MMSE, facial picture recognition test	Objective: to investigate the memory for facial identity in elderly depressive patients before and after treatment with escitalopram. Finding: escitalopram improved mood, overall cognitive performance, and the memory for negative facial stimuli, but not for positive stimuli.
		Clinical trial				
		*Depression*				
Barch et al. (2012)	MDD (DSM-IV) mean MADRS 26.2	68.1 (7.1)	Sertraline 50–200 mg (*n* = 166)	12 weeks	WRT, ROCFT, BVRT, DS-f, DS-b, DS-ascending	Objective: to examine the degree to which cognitive function improved during 12 weeks of sertraline treatment in older adults with MDD. Finding: episodic memory and executive function improved.
Devanand et al. (2003)	Outpatients with MDD, dysthymic disorder or depression NOS and cognitive impairment without dementia (DSM-IV) (mean HAM-D 15.4, mean MMSE 25.7)	72.0 (10.2)	Sertraline 50–200 mg (*n* = 26)	12 weeks	MMSE, DS-f, DS-b, WRT, DSST	Objective: to assess performance on tests of attention, executive function, memory, and language between responders and non-responders of sertraline on depression. Finding: responders improved more on DSST, but there were no differences in other measures of memory.
Diaconescu et al. (2011)	MDD non-bipolar, non-psychotic (DSM-IV) mean HAM-D 25.6	65.5 (9.1)	Citalopram 20–40 mg (*n* = 16)	8 weeks	Dementia Rating Scale, WRT	Objective: to identify the networks associated with improvement in affective symptoms and cognitive function during antidepressant treatment. Finding: patients showed improvement in affective and cognitive function as well as changes in cerebral glucose metabolism.
Nebes et al. (1999)	In- and out-patients with MDD non-bipolar, non-psychotic (DSM-IV) mean HAM-D 20.9	70.7 (6.4), 61–84	Paroxetine 10–40 mg (*n* = 29)	6 weeks	WRT, DSST	Objective: to examine cognitive changes in geriatric depressed patients during acute treatment with paroxetine. Finding: acute treatment with paroxetine was not associated with any cognitive impairment.
Rocca et al. (2005)	Minor depressive disorder or subsyndromal depressive symptomatology (DSM-IV) mean HAM-D 12.9	72.4 71.9	Citalopram 20 mg (*n* = 66) Sertraline 50 mg (*n* = 72)	1 year	WMS, MMSE	Objective: to compare over 1 year the effect of sertraline and citalopram on depressive symptoms and cognitive functions of non-demented elderly patients with depressive disorder NOS. Finding: sertraline and citalopram at low doses are equivalent in reducing depressive symptomatology and improvements of cognitive functions were observed with both treatments.
		*Other medical conditions*				
Royall et al. (2009)	Non-depressed outpatients with ischemic cerebrovascular disease	78.2 (7.2), 60–90	Sertraline 200 mg (*n* = 35)	6 weeks	MMSE	Objective: to study the effect of sertraline on isolated executive control function impairments in patients with ischemic cerebrovascular disease, as a prelude to future placebo-controlled clinical trials. Finding: sertraline may have significant effects on executive control function in cognitive impairment of dementia patients representing a subset of patients with vascular cognitive impairment.
		Cohort study				
		*Depression*				
Korten et al. (2014)	In- and out-patients with MDD, minor depression or dysthymia (*DSM*-IV, mean IDS score 30.1) and non-depressed older adults	70.7 (7.4)	Use of SSRI (*n* = 509)	N/A	WRT, DS-f, DS-b	Objective: to replicate the association between late-life depression and several domains of cognitive functioning and to examine which clinical characteristics of depression contribute independently to poorer cognitive function in late-life depression. Finding: depressed older adults have poorer cognitive functioning. A higher severity of psychopathological symptoms, having a first depressive episode, and the use of TCAs, SNRIs and benzodiazepines was associated with worse cognitive performance.
		*Dementia*				
Brendel et al. (2018)	Patients with mild cognitive impairment or Alzheimer’s dementia, depressed (based on positive score on NPI-Q item on depressive symptoms, mean MMSE 27.6) and non-depressed (mean MMSE 27.5)	71.6 (9.3)	Use of SSRI (*n* = 24)	2 years	Alzheimer’s Disease Assessment Scale	Objective: to investigate the influence of SSRI use on longitudinal neuroimaging findings of amyloid load and brain volume in conjunction with cognitive assessment. Finding: subjects with depressive symptoms were characterized by faster cognitive decline and faster progression of gray matter atrophy if not receiving SSRI treatment.
Oh et al. (2021)	Patients with probable Alzheimer dementia (mean MMSE 21.3)	75.5 (9.8)	Use of SSRI (*n* = 2274)	1 year	MMSE	Objective: to determine if exposure to antidepressants, antipsychotics, and benzodiazepines in AD would be associated with poorer cognitive, functional, or neuropsychiatric outcomes over time. Finding: exposure to atypical antipsychotics was associated with more rapid decline in cognition and increase in dementia severity, and non-SSRI depressants were associated with slower rate of increase in dementia severity.
Pirker-Kees et al. (2019)	Outpatients with dementia (mean MMSE 21)	76 (8.8)	Use of SSRI (*n* = 22)	12 months	MMSE	Objective: to evaluate the effect of psychotropic medication on cognition, behavioral symptoms, and caregiver burden in AD. Finding: MMSE and caregiver burden increased significantly over time, but none of the individual medication groups changed significantly over time.
Rozzini et al. (2010)	Outpatients with probable AD (mean MMSE 20.3), with and without depression	76.7 (6.7)	Use of SSRI (*n* = 66)	9 months	MMSE	Objective: to evaluate the role on cognition of combining SSRIs with medication used in AD, hypothesizing a joint effect that may delay cognitive deterioration. Finding: use of SSRI in people with AD treated with AChEIs may exert some degree of protection against the negative effect of depression on cognition.
		*General population*				
Carrière et al. (2017)	Community-dwelling persons	74, 70–78	Use of SSRI (*n* = 296)	10 years	BVRT, MMSE	Objective: to prospectively examine the association between antidepressant use and 10-year decline in five cognitive domains in a large elderly community-dwelling cohort. Finding: antidepressant use was not significantly associated with cognitive decline.
Del Ser et al. (2019)	Community-dwelling persons	74.7 (3.9)	Use of SSRI (*n* = 103)	2 years	MMSE, WRT, DSST	Objective: to examine in a cohort of cognitively normal older adults the eventual deleterious or positive effects on psychometric performance of drugs most commonly prescribed in the elderly population. Finding: only a few drugs show a very small cognitive effects and only on information processing.
Leng et al. (2018)	Community-dwelling women	83.4 (2.8)	Use of SSRI (*n* = 77)	5 years	MMSE	Objective: to investigate the relationship between the use of different antidepressants and change in cognitive function. Finding: those who were using SSRIs had the greatest decline in cognitive function over 5 years, compared to those not taking any antidepressants and those who were taking other types of antidepressants.

SD: standard deviation; *n*: sample; MDD: major depressive disorder; DSM: Diagnostic and Statistical Manual of Mental Disorders; N/A: not available; mg: milligram; MMSE: mini-mental state examination; DSST: Digit Symbol Substitution Test; SLT: Shopping List Task; ICD: International Classification of Diseases; WRT: Word Recall Test; SSRI: selective serotonin reuptake inhibitor; WAIS: Wechsler Adult Intelligence Scale; FTD: frontotemporal dementia; DS: digit span; CANTAB: Cambridge Neuropsychological Test Automated Battery; AD: Alzheimer’s disease; NINCDS/ADRDA: National Institute for Neurological and Communicative Diseases and Stroke/Alzheimer’s Disease and Related Disorders Association; RBMT: Rivermead Behavioral Memory Test; ADAS: Alzheimer’s Disease Assessment Scale; WMS: Wechsler Memory Scale; PST: problem-solving therapy; RBANS: Repeatable Battery for the Assessment of Neuropsychological Status; HAM-D: Hamilton Rating Scale for Depression; rTMS: repetitive Transcranial Magnetic Stimulation; PD: personality disorder; HAM-A: Hamilton Rating Scale for Anxiety; FIM: Functional Independence Measure; CAMCOG: Cambridge Cognitive Examination; MADRS: Montgomery Asberg Depression Rating Scale; ROCFT: Rey–Osterrieth Complex Figure Test; BVRT: Benton Visual Retention Test; NOS: not otherwise specified; CNS: central nervous system; NIH: National Institutes of Health; GDS: Geriatic Depression Scale; GAD: generalized anxiety disorder; IDS: Inventory of Depressive Symptomatology; TCA: tricyclic antidepressants; SNRI: serotonin-norepinephrine reuptake inhibitor; NPI-Q: Neuropsychiatric Inventory Questionnaire.

The risk of bias for the included studies varied from low risk of bias (*n* = 20) to serious risk of bias (*n* = 3). Especially in the non-randomized studies, there was moderate to serious bias due to confounding, but also in the randomized controlled trials the randomization process was sometimes not described clearly (e.g. method of randomization not specified) (Table 2).

**Table 2.** Risk of bias.

Author (year)	Low risk of bias	Moderate risk of bias	Serious risk of bias
*Randomized controlled trial*			
Boggio et al. (2005)	X		
Bondareff et al. (2000)	X		
Cassano et al. (2002)		X	
Culang et al. (2009)	X		
Geretsegger et al. (1994)		X	
Newhouse et al. (2000)	X		
Raskin et al. (2007)		X	
Taragano et al. (1997)		X	
Choe et al. (2016)	X		
Deakin et al. (2004)		X	
Mokhber et al. (2014)	X		
Munro et al. (2004)		X	
Munro et al. (2012)			X
Porsteinsson et al. (2014)	X		
Weintraub et al. (2010)	X		
Martin et al. (1989)			X
O’Carroll et al. (1994)		X	
Butters et al. (2011)	X		
Jorge et al. (2010)	X		
Robinson et al. (2000)		X	
Furlan et al. (2001)	X		
Kerr et al. (1992)		X	
Lenze et al. (2020)	X		
*Controlled clinical trial*			
Alves et al. (2007)		X	
Beheydt et al. (2015)		X	
Savaskan et al. (2008)		X	
*Clinical trial*			
Barch et al. (2012)		X	
Devanand et al. (2003)		X	
Diaconescu et al. (2011)		X	
Nebes et al. (1999)	X		
Rocca et al. (2005)	X		
Royall et al. (2009)	X		
*Cohort study*			
Korten et al. (2014)	X		
Brendel et al. (2018)		X	
Oh et al. (2021)	X		
Pirker-Kees et al. (2019)	X		
Rozzini et al. (2010)	X		
Carrière et al. (2017)			X
Del Ser et al. (2019)		X	
Leng et al. (2018)	X		

### Effects on global memory

In total, 29 studies assessed global memory, using MMSE, WMS, Blessed Information and Memory Test (BIMT), the Clifton Assessment Schedule (CLAS), Alzheimer’s Assessment Scale (ADAS), the Rivermead Behavioral Memory Test (RBMT), the Repeatable Battery for the Assessment of Neuropsychological Status (RBANS), the Cambridge Cognitive Examination (CAMCOG), the Dementia Rating Scale (DRS) or the National Institutes of Health (NIH) Toolbox Cognition Battery, and Alzheimer’s Disease Assessment Scale. Of these, 11 studies reported an association between SSRI use and improvement of global memory functioning, 16 studies reported no effect, and 2 studies reported an impairment in global memory functioning associated with SSRI use. The results are shown in Table 3, specified per type of memory.

**Table 3.** Results of studies, divides into different types of memory.

Author (year)	Global memory	Episodic memory	Visual/spatial memory	Short-term memory	Working memory
*Randomized controlled trial*					
Boggio et al. (2005)					0
Bondareff et al. (2000)	+	+		+	+
Cassano et al. (2002)	+	+			
Culang et al. (2009)		0/−*			0/−*
Geretsegger et al. (1994)	+				
Newhouse et al. (2000)		+			+
Raskin et al. (2007)	0	+		+	0
Taragano et al. (1997)	0				
Choe et al. (2016)	0				
Deakin et al. (2004)		–		0	0
Mokhber et al. (2014)	+				
Munro et al. (2004)	0	0		0	
Munro et al. (2012)	0				0
Porsteinsson et al. (2014)	–				
Weintraub et al. (2010)	0				
Martin et al. (1989)	+	+			
O’Carroll et al. (1994)	0			0	0
Butters et al. (2011)		0		0	0
Jorge et al. (2010)	+	+		+	
Robinson et al. (2000)	0				
Furlan et al. (2001)		0		0	0
Kerr et al. (1992)				0	
Lenze et al. (2020)	+				
*Controlled clinical trial*					
Alves et al. (2007)	+	+		0	
Beheydt et al. (2015)		0		0	0
Savaskan et al. (2008)	+	+			
*Clinical trial*					
Barch et al. (2012)		+		0	0
Devanand et al. (2003)	0	0		0	+/−*
Diaconescu et al. (2011)	0	+			
Nebes et al. (1999)		0		0	+
Rocca et al. (2005)	+				
Royall et al. (2009)	0				
*Cohort study*					
Korten et al. (2014)		0			0
Brendel et al. (2018)	+				
Oh et al. (2021)	0				
Pirker-Kees et al. (2019)	0				
Rozzini et al. (2010)	0*				
Carrière et al. (2017)	0		0		
Del Ser et al. (2019)	0	0			0
Leng et al. (2018)	–				

0: no effect; −: impairment; +: improvement.

* Results depend on responding status: responders versus non-responders.

Improvement of global cognition between baseline and 3 months, but at 9 months, stabilization from baseline.

### Effects on episodic memory

Episodic memory functioning was assessed using Word Recall Test (WRT), Shopping List Task (SLT), facial picture recognition test, delayed pattern recognition, paired-associate learning (PAL), and subtests of the RBANS and CAMCOG. Half of these studies (10 out of 20) found an improvement in episodic memory functioning, while 8 studies reported no effect. One study found the effect to be depended on responding status. Patients who improved on depression after use of SSRI did not experience an effect on episodic memory, but patients who did not improve on depression did experience an impairment in episodic memory functioning. One other study reported a post-SSRI impairment in episodic memory functioning, measured with delayed pattern recognition task (see Table 3).

### Effects in visual or spatial memory

One study has assessed visual or spatial memory, using the Benton Visual Retention Test. This prospective cohort study did not find an effect of SSRIs on visual memory (see Table 3).

### Effects on short-term memory

Fourteen studies assessed the effect of SSRIs on short-term memory, using forward digit span, consonant trigrams, Wechsler logical memory, immediate recall of WRT, SLT, PAL, and pattern recognition and subtests of RBANS, WMS, and CAMCOG. Three reported an improvement, while 11 studies reported no effect. No studies reported an impairment of short-term memory after use of SSRI (see Table 3).

### Effects on working memory

Working memory was assessed in 16 studies, using the Digit Symbol Substitution Test (DSST), backwards digit span, letter–number sequencies, and the Wechsler figural memory. In 3 studies, an improvement in working memory functioning was found, while in 11 studies, no effect was reported. In 2 studies, the effect of SSRIs on working memory depended on responding status; responders showed no effect in one study and an improvement in the other study. In both studies, non-responders showed impairment of working memory after use of SSRI (see Table 3).

### Effects on memory in depressed subjects

In 17 studies, the effects of SSRIs on memory functioning in older depressed subjects were reported. The results are shown in Supplemental Appendix, Table 4. Global memory was assessed in 10 studies, of which 6 showed improvement while the others reported no effect after SSRI treatment. Episodic memory improved after use of SSRI in 8 of 13 studies, 4 studies found no effect, and 1 study found the effect to be depended on responding status (no effect in responders and impairment in non-responders). Short-term memory was assessed in seven studies, two showing improvement and five did not report any effect. The effects of SSRIs on working memory was depended on responding status according to two studies: one found that responders did not have any effect while non-responders experienced impairment and the other study reported an improvement in responders and an impairment in non-responders. Three studies showed an improvement in working memory functioning after SSRI use and the remaining five studies found no effect. The quality of the studies in depressed subjects was good to moderate (Table 2).

### Effects on memory in subjects with dementia

Thirteen studies included subjects with dementia of various etiologies (Supplemental Appendix, Table 5). Global memory was assessed in 12 of them; 3 reported improvement, 1 reported impairment, and the remaining 8 studies reported no effect on memory functioning. Episodic memory was measured in three studies, one reporting improvement, one reporting impairment, and one reporting no effect. Three studies reported on the association between short-term memory and working memory and SSRI use in patients with dementia, but none found an association. Most studies in dementia patients were of good quality, except two studies had serious risk of bias (Martin et al., 1989; Munro et al., 2012) (Table 2).

### Effects on memory in general population

Five studies of good to moderate quality included healthy subjects from the general population. The results are shown in Supplemental Appendix, Table 6. Two studies reported outcomes on global memory functioning, of which one study found a positive effect and the other found no effect. Episodic memory, short-term memory, and working memory were reported in two studies, none of them reported an effect.

### Effects on memory in miscellaneous study populations

We grouped five studies as “miscellaneous.” Post-stroke cognitive functioning related to use of SSRI was reported in three studies (Jorge et al., 2010; Robinson et al., 2000; Royall et al., 2009), one study investigated older adults with generalized anxiety disorder (GAD) (Butters et al., 2011), and one study a population of community-dwelling older adults who used antidepressants (Carrière et al., 2017). The results are shown in Supplemental Appendix, Table 7. In post-stroke patients, improvement of global memory, episodic memory, and short-term memory was reported in one study, while the other two studies reported no effect on global memory functioning. In older adults with GAD, no effects were reported on episodic memory, short-term memory, or working memory. In community-dwelling older adults who used antidepressants, no effects were found on global memory or visual memory, but this study had serious risk of bias (Carrière et al., 2017).

## Discussion

The aim of the present systematic review was to summarize all relevant findings on the effects of SSRIs on all aspects of memory function in older adults.

Overall, no consistent negative effects of SSRIs on memory functioning in older adults were found after 4 weeks to 10 years of SSRI treatment. The majority of studies reported no change in memory functioning after SSRI use. Interestingly, also positive effects of SSRIs on memory functioning were found. This seemed to be most pronounced in older adults with mental or neurological disorders, such as subjects with depression or stroke.

### Depression

Global memory and episodic memory functioning in patients with depression seemed to be most sensitive to positive influence of SSRI use (see Table 4). The relationship between improved memory functioning and improved depression symptoms (treatment effect) is difficult to determine on basis of the current review. However, two studies reported that improvement of memory functioning was associated with responder status (Culang et al., 2009; Devanand et al., 2003). Non-responders were more likely to experience impairment in memory functioning, while responders showed no effect (Culang et al., 2009) or even improvement (Devanand et al., 2005). In total, three studies found correlations between (remission of) depression and cognitive outcome (Culang et al., 2009; Devanand et al., 2003; Diaconescu et al., 2011). In contrast, other studies have reported that the effect was independent of depression symptoms (Jorge et al., 2010; Raskin et al., 2007). Also, a greater reduction in depression scores did not predict a greater effect on memory functioning (Barch et al., 2012). Baseline severity of depression did not seem to influence results. The majority of studies were conducted in patients with severe depression, but also in mild-to-moderate depressions, some studies reported improvement of memory functioning (Rocca et al., 2005; Savaskan et al., 2008) and others no effect (Devanand et al., 2003; Korten et al., 2014). Taken together, the association between improved memory performance and depression conditions cannot be ascertained on basis of the current review.

Also, in some of the studies in depressed patients, two treatments or SSRIs were compared to each other (Alves et al., 2007; Bondareff et al., 2000; Cassano et al., 2002; Furlan et al., 2001; Geretsegger et al., 1994; Mokhber et al., 2014; Newhouse et al., 2000; Robinson et al., 2000; Rocca et al., 2005; Taragano et al., 1997). Inherent to this study design is the possible bias of practice effects, meaning that the improvement reported is not due to treatment, but to the fact that patients learn to perform better on the cognitive tasks. Even though practice effect is an important factor to take into account, in this review also placebo-controlled studies and studies with a healthy comparison group showed positive effects of SSRIs on memory functioning (Alves et al., 2007; Jorge et al., 2010; Lenze et al., 2020; Raskin et al., 2007; Savaskan et al., 2008), making it unlikely that practice effect accounts for all reported improved memory functioning.

### Dementia and other brain diseases

Results in patients with dementia were mostly pointing toward no effect of SSRI use on memory performance, but three studies indicated possible improvement of memory functioning (Brendel et al., 2018; Martin et al., 1989; Mokhber et al., 2014) (see Table 5 in Supplemental Appendix). Interestingly, two studies reported impairment of memory functioning associated with SSRI use (Deakin et al., 2004; Porsteinsson et al., 2014). Both studies were performed in dementia with frontal dysfunction. This underlying subtype could potentially be more prone to SSRI-associated memory impairment.

In post-stroke patients, Jorge et al. (2010) reported improvement of global memory, episodic memory, and short-term memory. Two other studies failed to replicate this finding in global memory functioning (Robinson et al., 2000; Royall et al., 2009).

### Healthy controls

Five studies assessed the association between SSRI use and memory functioning in healthy older adults (Carrière et al., 2017; Del Ser et al., 2019; Furlan et al., 2001; Kerr et al., 1992; Lenze et al., 2020; Leng et al., 2018) (see Table 6 in Supplemental Appendix). The majority reported no association. Lenze et al. (2020), however, reported an improvement in global memory functioning in older adults who did not have objective cognitive deficits, but did experience subjective cognitive decline. This could represent an early cognitive vulnerability. One study did report an impairment in global memory functioning (Leng et al., 2018). Interestingly, this study did only include women which could indicate a gender effect.

### Other factors that may have affected results

The studies that were included in which SSRI treatment lasted 12 weeks or longer (Barch et al., 2012; Beheydt et al., 2015; Bondareff et al., 2000; Brendel et al., 2018; Butters et al., 2000, 2011; Carrière et al., 2017; Cassano et al., 2002; Choe et al., 2016; Del Ser et al., 2019; Devanand et al., 2003; Jorge et al., 2010; Leng et al., 2018; Lenze et al., 2020; Mokhber et al., 2014; Munro et al., 2004, 2012; Newhouse et al., 2000; Oh et al., 2021; Pirker-Kees et al., 2019; Robinson et al., 2000; Rocca et al., 2005; Rozzini et al., 2010; Weintraub et al., 2010) were slightly more likely to find positive effects than studies reporting results of treatments of 4 weeks or shorter (Deakin et al., 2004; Furlan et al., 2001; Kerr et al., 1992; Martin et al., 1989; O’Carroll et al., 1994; Savaskan et al., 2008) and less likely to find negative results. This could indicate that long-term SSRI use does not have a deleterious effect on brain functions related to memory.

While our results did not show any convincing evidence of a negative effect of SSRI use on memory functioning in older adults, there are some contradictory findings in other studies with adult volunteers. It is suggested that SSRI use could lead to impaired memory functioning in adult volunteers; negative results on learning, episodic memory, and short-term memory have been reported (Chamberlain et al., 2006; Schmitt et al., 2001; Skandali et al., 2018; Wadsworth et al., 2005). It should be noted that these acute effects may be dose-dependent (Saletu et al., 1986). Together with the long-term studies reported in this review, this suggests that SSRI treatment can have an initial deleterious effect on memory but after reaching a steady state, these effects are no longer found. This difference can be explained on basis of the difference of acute- and long-term treatment of SSRIs on the brain (Sugrue, 1983).

Another observation was that underlying neuropsychiatric vulnerability appeared to play a role in the effect of SSRI use on memory functioning. This is also suggested by findings of a negative effect of acute tryptophan depletion on global memory (measured by MMSE) only in patients with Alzheimer’s disease and remitted depression, but not in healthy older control subjects (Porter et al., 2000, 2005) (although it should be noted that completing the MMSE is a fairly easy task for most healthy older adults, which could lead to failure to notice a more subtle impairment). A possible explanation for these findings is the hypothesis that memory performance relates to 5-HT in an inverted U-curve, implicating that memory performance is optimal at a certain level of 5-HT, but decreases when 5-HT levels increase or decrease from that optimum (Meeter et al., 2006; Riedel et al., 1999, 2002). Considering impaired 5-HT functioning as an important pathogenic factor in both depression (Jacobsen et al., 2012) and Alzheimer’s disease (Xu et al., 2012), it can be understood why patients with these neuropsychiatric disorders are more likely to experience cognitive improvement with SSRI treatment, compared to healthy subjects who are already on the 5-HT optimum.

### Methodologic considerations

The strength of this systematic review is the inclusion of studies using study designs of sufficient quality with a broad range of study populations. Nevertheless, there are a number of limitations that needs to be mentioned. The present findings must be interpreted with caution, because there is always a risk of publication bias. Some studies included a small number of patients, which can lead to low statistical power and failure to identify an effect of SSRI treatment (Type II error). There did not seem to be a difference in reported results between studies with small populations versus large populations. Also, the studies used different outcome measures to test memory performance, making direct comparisons of findings difficult. Some tests may be more sensitive for change after treatment with an SSRI. There was heterogeneity in study population and intervention (type of SSRI). Therefore, we also reported the results both for the total study population and per subgroups.

In conclusion, the current systematic review showed that SSRIs do not impair memory performance in older adults and patients. Interestingly, some subgroups may even benefit from long-term SSRI treatment on memory performance. For refining these subgroups, further research is recommended. A randomized placebo-controlled cross-over design in older adults with and without neuropsychiatric vulnerability could be recommended. Long-term effects, possible confounding factors, such as depressive complaints, pre-existing cognitive impairment, gender, and variability in type and dose of SSRI, but also clinically relevant outcome measurements such as quality of life should be taken into account.

## Supplemental Material

sj-docx-1-jop-10.1177_02698811221080462 – Supplemental material for The effects of selective serotonin reuptake inhibitors on memory functioning in older adults: A systematic literature review Click here for additional data file. Supplemental material, sj-docx-1-jop-10.1177_02698811221080462 for The effects of selective serotonin reuptake inhibitors on memory functioning in older adults: A systematic literature review by Julie EM Schulkens, Kay Deckers, Maud Jenniskens, Arjan Blokland, Frans RJ Verhey and Sjacko Sobczak in Journal of Psychopharmacology
