# Supplementary material for: The effects of selective serotonin reuptake inhibitors on memory functioning in older adults: A systematic literature review
Source: J Psychopharmacol. 2022 Apr 29;36(5):578–93. doi: 10.1177/02698811221080462 (PMC9112622; doi:10.1177/02698811221080462)
Supplement: sj-docx-1-jop-10.1177_02698811221080462 – Supplemental material for The effects of selective serotonin reuptake inhibitors on memory functioning in older adults: A systematic literature review [file sj-docx-1-jop-10.1177_02698811221080462.docx]

**Appendix 1: complete search strategy**

*PubMed*

S1: ((((((Selective serotonin reuptake inhibitor) OR Serotonin reuptake inhibitor) OR Serotonin Uptake inhibitor) OR SSRI) OR "Serotonin Uptake Inhibitors"[Mesh]))

S2: Memory OR "Memory"[Mesh] OR Working memory OR "Memory, Short-Term"[Mesh] OR Short term memory OR Short-term memory OR Long term memory OR Long-term memory OR "Memory, Long-Term"[Mesh] OR Spatial memory OR "Spatial Memory"[Mesh] OR Neuropsychology OR "Neuropsychology"[Mesh] OR "Cognition"[Mesh] OR Cognition OR Cognitive OR cognitive performance OR cognitive ability OR cognitive abilities

S3: S1 AND S2

S4: limit S3 to ’Humans’ and ‘English language’ and ‘Aged: 65+ years’ (filters)

18.10.2021: 622 hits

*PsycINFO*

S1: ( Serotonin Reuptake Inhibitors" OR Selective serotonin reuptake inhibitors OR SSRI OR Serotonin reuptake inhibitors OR Serotonin uptake inhibitors )

S2: ( Memory OR "Memory, Short-term" OR ( short term memory or working memory or immediate memory ) OR ( long term memory or long-term memory or long term memory retrieval ) OR Spatial memory OR "Neuropsychology" OR ( neuropsychology or neuropsychological ) OR "Cognition" OR ( cognition or cognitive function or cognitive performance or cognitive abilities or cognitive ability)

S3: S1 AND S2

S4: limit S3 to ‘Aged (65 yrs & older) and ‘English language’

18.10.2021: 201 hits

*CINAHL*

S1: "( Serotonin Uptake Inhibitors" OR Selective serotonin reuptake inhibitors OR SSRI OR Serotonin reuptake inhibitors OR Selective serotonin uptake inhibitors )

S2: ( Memory OR "Memory, Short-term" OR ( short term memory or working memory or immediate memory ) OR ( long term memory or long-term memory or long term memory retrieval ) OR Spatial memory OR "Neuropsychology" OR ( neuropsychology or neuropsychological ) OR "Cognition" OR ( cognition or cognitive function or cognitive performance or cognitive abilities or cognitive ability)

S3: S1 AND S2

S4: limit S3 to ‘English language’ and ‘Aged: 65+ years’

18.10.2021: 109 hits

*Embase*

S1: (Serotonin Uptake Inhibitor or Selective Serotonin Reuptake Inhibitor or Serotonin Reuptake Inhibitor or Selective Serotonin Uptake Inhibitor or SSRI)

S2: Memory or Spatial Memory or Long Term Memory or Short Term Memory or Working Memory or Neuropsychology or Neuropsychological or Cognition or Cognitive or Cognitive performance or Cognitive ability or Cognitive abilities

S3: S1 AND S2

S4: limit S3 to ‘Human’ and ‘English language’ and ‘Aged <65+ years>’

18.10.2021: 956 hit

**Appendix 2: results on the effects of SSRI use on memory functioning divided into different types of memory for each studied population**

Table 4 - Results of studies that included currently depressed subjects (n=17)

| Author (year) | Global memory | Episodic memory | Visual/spatial memory | Short-term memory | Working memory |
| --- | --- | --- | --- | --- | --- |
| *Randomized Controlled Trial* | | | | | |
| Boggio et al. (2005) |  |  |  |  | 0 |
| Bondareff et al. (2000) | + | + |  | + | + |
| Cassano et al. (2002) | + | + |  |  |  |
| Culang et al. (2009) |  | 0/- * |  |  | 0/- * |
| Geretsegger et al. (1994) | + |  |  |  |  |
| Newhouse et al. (2000) |  | + |  |  | + |
| Raskin et al. (2007) | 0 | + |  | + | 0 |
| Taragano et al. (1997) | 0 |  |  |  |  |
| *Controlled Clinical Trial* | | | | | |
| Alves et al. (2007) | + | + |  | 0 |  |
| Beheydt et al. (2015) |  | 0 |  | 0 | 0 |
| Savaskan et al. (2008) | + | + |  |  |  |
| *Clinical Trial* | | | | | |
| Barch et al. (2012) |  | + |  | 0 | 0 |
| Devanand et al. (2003) | 0 | 0 |  | 0 | +/- * |
| Diaconescu et al. (2011) | 0 | + |  |  |  |
| Nebes et al. (1999) |  | 0 |  | 0 | + |
| Rocca et al.(2005) | + |  |  |  |  |
| *Cohort study* | | | | | |
| Korten et al. (2014) |  | 0 |  |  | 0 |

*0 = no effect; - = impairment; + = improvement;  ^*^results depend on responding status: responders vs non-responders*

*Table 5 - Results of studies that included subjects with a form of dementia (n=13)*

| Author (year) | Global memory | Episodic memory | Visual/spatial memory | Short-term memory | Working memory |
| --- | --- | --- | --- | --- | --- |
| *Randomized Controlled Trial* | | | | | |
| Choe et al. (2016) | 0 |  |  |  |  |
| Deakin et al. (2004) |  | - |  | 0 | 0 |
| Mokhber et al. (2014) | + |  |  |  |  |
| Munro et al. (2004) | 0 | 0 |  | 0 |  |
| Munro et al.(2012) | 0 |  |  |  | 0 |
| Porsteinsson (2014) | - |  |  |  |  |
| Weintraub et al. (2010) | 0 |  |  |  |  |
| Martin et al. (1989) | + | + |  |  |  |
| O’Carroll et al. (1994) | 0 |  |  | 0 | 0 |
| *Cohort study* | | | | | |
| Brendel et al. (2018) | + |  |  |  |  |
| Oh et al. (2021) | 0 |  |  |  |  |
| Pirker-Kees et al. (2019) | 0 |  |  |  |  |
| Rozzini et al. (2010) | 0* |  |  |  |  |

*0 = no effect; - = impairment; + = improvement ** Improvement of global cognition between baseline and 3 months, but at 9 months stabilization from baseline.

*Table 6 – Results of studies that are conducted in the general population of older adults (n=5)*

| Author (year) | Global memory | Episodic memory | Visual/spatial memory | Short-term memory | Working memory |
| --- | --- | --- | --- | --- | --- |
| *Randomized Controlled Trial* | | | | | |
| Furlan et al. (2001) |  | 0 |  | 0 | 0 |
| Kerr et al. (1992) |  |  |  | 0 |  |
| Lenze et al. (2020) | + |  |  |  |  |
| *Cohort study* | | | | | |
| Del Ser (2019) | 0 | 0 |  |  | 0 |
| Leng et al. (2018) | - |  |  |  |  |

*0 = no effect; - = impairment; + = improvement;*

*Table 7 – Results of the study that included other groups: subjects with a stroke (n=3), subjects with generalized anxiety disorder (n=1) and community dwelling persons who use antidepressants (n=1)*

| Author (year) | Global memory | Episodic memory | Visual/spatial memory | Short-term memory | Working memory |
| --- | --- | --- | --- | --- | --- |
| *Randomized Controlled Trial* | | | | | |
| Butters et al. (2011) |  | 0 |  | 0 | 0 |
| Jorge et al. (2010) | + | + |  | + |  |
| Robinson et al. (2000) | 0 |  |  |  |  |
| *Clinical Trial* | | | | | |
| Royall et al. (2009) | 0 |  |  |  |  |
| *Cohort study* | | | | | |
| Carrière et al. (2017) | 0 |  | 0 |  |  |
